# Nonsteroidal anti-inflammatory drug administration to periparturient cows to reduce postpartum pain-related behaviours

**DOI:** 10.18849/ve.v9i4.692

**Published:** 2024-10-18

**Authors:** Amelia Cannadine

**Affiliations:** University of Sydney, Australia

**Keywords:** behaviour, cows, heifers, nsaids, pain, pain-relief, parturition, welfare

## Abstract

**PICO question:**

In periparturient cows does the administration of nonsteroidal anti-inflammatory drugs (NSAIDs) aid in reducing pain-related behaviours after parturition when compared to cows not administered NSAIDs?

**Clinical bottom line:**

**Category of research:**

Treatment.

**Number and type of study designs reviewed:**

Four randomised control trials.

**Strength of evidence:**

Moderate.

**Outcomes reported:**

The evidence suggests that NSAIDs can reduce some pain-related behaviours and increase resting behaviours in postpartum cows, with the effect being most consistently observed when administered between 6 and 48 hours before calving or within 3 hours after calving. This effect was most consistently observed in individuals that had experienced uncomplicated calving events and primiparous animals. Primiparous animals administered meloxicam prior to natural calving displayed significantly more lying bouts on the day of calving when compared to primiparous control animals and primiparous animals administered meloxicam postpartum. A separate study reported that cows administered ketoprofen spent less time in lateral recumbency than cows in the placebo group, irrespective of whether calving was assisted. Additionally, when treatment cows were in sternal recumbency, they spent more time with their head in a rested position than the placebo group. There was no significant difference in feeding behaviours postpartum between treatment and placebo animals. There were conflicting results between papers assessing activity levels.

**Conclusion:**

In some cows, in particular cows with an uncomplicated parturition, NSAIDs can reduce some pain-related behaviours and increase some resting behaviours postpartum. The NSAID should be administered between 6 and 48 hours before calving or within 3 hours after calving.

## How to apply this evidence in practice

The application of evidence into practice should take into account multiple factors, not limited to: individual clinical expertise, patient’s circumstances and owners’ values, country, location or clinic where you work, the individual case in front of you, the availability of therapies and resources.

Knowledge Summaries are a resource to help reinforce or inform decision-making. They do not override the responsibility or judgement of the practitioner to do what is best for the animal in their care.

## Clinical scenario

In recent decades, awareness around the importance of nonsteroidal anti-inflammatory drugs (NSAIDs) in animals has increased dramatically (Edwards, 2021). NSAIDs have been reported to be efficacious at reducing pain inflicted on cattle during husbandry procedures, such as castration, dehorning, and disbudding (Steagall et al., 2021). However, it is not only these procedures that have proved to induce pain in cattle. The literature has demonstrated the presence of an inflammatory state in postpartum cows from the tissue trauma associated with calving, even in healthy individuals (Humblet et al., 2006; Trimboli et al., 2020).

To date, the effects of postpartum analgesia on production parameters has been the focus in existing literature (Carpenter et al., 2016). However, very few studies include measures more typically considered to be sensitive indicators of welfare status, such as behaviour. Lying, for example, is a biologically important behaviour associated with sleeping and ruminating which can be reduced if an animal is experiencing stress, and potentially pain can have a similar effect (Gladden et al., 2021; Ruckebusch, 1975; Tucker et al., 2021). Given that pain is a primary welfare concern, determining whether the provision of immediate postpartum analgesia provides welfare benefits is essential, particularly in primiparous cows or in cows experiencing, or expected to experience, varying severities of dystocia (Gladden et al., 2021). Thus, evaluation of the evidence that measures behavioural parameters in periparturient cows administered NSAIDs is required to inform veterinarians and producers whether such a protocol can optimise welfare in the breeding herd.

## The evidence

The evidence consists only of randomised control trials, which are ranked as high-level evidence due to their unbiased design and minimal risk of systematic errors (Burns et al., 2011; Sargeant et al., 2022). The evidence is weakened by those studies that omitted evidence of sample size calculations or 95% confidence, which would have evaluated whether sample size was sufficient, thus we cannot confirm whether there were enough study subjects to reduce type two errors (Jones et al., 2003; Mainau et al., 2014; Swartz et al., 2018). Furthermore, the variation in methodology between the papers limits the ability to compare them. Three NSAID types were utilised amongst the studies, namely, meloxicam (Mainau et al., 2014; Swartz et al., 2018), ketoprofen (Gladden et al., 2021), and acetylsalicylic acid (Barragan et al., 2020). Only one study included administration of an NSAID prior to calving as part of their protocol (Swartz et al., 2018), whilst administration post-calving was conducted in four of the studies (Barragan et al., 2020; Gladden et al., 2021; Mainau et al., 2014; Swartz et al., 2018). The studies containing the greatest number of significant findings for the reduction in pain-related behaviours and the promotion of resting behaviours came from those cows that were administered an NSAID prior to or promptly after parturition.

## Summary of the evidence

**Barragan et al. (2020):** 

Population	Postpartum dairy cows (breed not reported) that did not experience a fetotomy or caesarean or were not diagnosed as having a health disorder in the first week of lactation were included in the study. This study was performed between May and July 2016 in three organic dairy farms in Colorado, USA, that milked 3,000–5,300 cows.
Sample Size	464 cows.
Intervention Details	Within 12 hours after parturition, cows were blocked by parity and calving ease and randomly assigned to one of two treatment groups: The treatment group (ASP) (n = 223) received 4 consecutive treatments every 12 hr with acetylsalicylic acid (100 mg/kg; 2 boluses). The placebo group (PLC) (n = 241) received 4 treatments every 12 hr with gelatin capsules (2 capsules) filled with water. At 21 ± 3 days prior to parturition, cows and pregnant heifers were moved to prepartum pens in a common maternity facility. The prepartum pens consisted of loose pens with wood shavings as bedding material and free access to a contiguous dry lot. Within one day of parturition, postpartum cows were moved to their respective farms. Fresh cows were housed on sand-bedded 6-row free stall barns with access to a contiguous dry lot. Space allowance per cow was not discussed.
Study design	Randomised control trial.
Outcome studied	Daily milk yield data was obtained from on-farm computer systems. Within each treatment, a subset of cows was randomly assigned to receive an activity logger accounting for parity (primiparous and multiparous) and calving ease (eutocia and dystocia). At enrolment, data loggers were placed on the right hind leg of a subset of cows for assessment of lying time (LT), number of steps (STP), number of lying bouts (LB), and lying bouts duration (LBD). The activity data loggers were removed 7 days in milk later (7 DIM). Blood samples were collected from coccygeal blood vessels at 7 ± 3 and 14 ± 3 DIM for assessment of beta-hydroxybutyrate (BHB) concentrations.
Main findings (relevant to PICO question)	A total of 16 cows (ASP = 9; PLC = 7) were not included in the study due to loss of follow up during treatment administration. Furthermore, cows diagnosed with having a health disorder in the first week of lactation were removed from the study. As a result, 464 animals remained in the study (223 in the treatment group and 241 in the placebo group). A smaller sample size was utilised for the activity tracking data (ASP = 36; PLC = 41) and it is unclear whether a power calculation or was performed for this smaller subset and there was no evidence of 95% confidence intervals to evaluate whether the smaller sample size was sufficient. Overall, there was no difference in LT, LB, and LBD between cows in the ASP and PLC groups. However, cows treated with aspirin tended to spend less time lying down and tended to have shorter LBD during the first day after parturition compared with PLC cows. But these findings were deemed insignificantly different (P > 0.05). Cows in the ASP group had more STP compared with cows in the PLC group.
Limitations	No mention of animal ethics approval. There was no mention of blinding of any personnel involved in the study. Accurate prediction of the degree of calving difficulty was not possible until the cow was in labour, so to assess activity patterns accounting for parity and calving ease, a subset of cows in each treatment group were randomly assigned to receive activity data loggers at the time of first treatment.

Table note...

**Gladden et al. (2021):** 

Population	Holstein dairy cows from a 700 dairy cow herd in Scotland were recruited (year not reported) provided that they were not lame, they had no signs of clinical illness at parturition, and they gave birth to a live female Holstein calf.
Sample Size	72 cows recruited.
Intervention Details	Following recruitment, cows were randomly allocated to either a treatment group or a placebo group (number in each group not stated) and administered either a single dose of ketoprofen (Ketaprofen 10%) at 3 mg/kg or a single dose of saline within 3 hours of parturition. Parity was also considered when allocation with similar numbers of primiparous and multiparous animals in each treatment group. 37 cows experiencing assistance at parturition (17 treatment and 20 placebo) and 35 cows not experiencing assistance at parturition (18 treatment and 17 placebo). Both treatments were administered by deep intramuscular injection within 3 hours of parturition. Initially cows were housed in cubicles before being moved to a straw-bedded group pen three weeks prior to expected parturition. Parturition occurred in this pen after which cows were moved to an adjacent postpartum pen, or before, the next milking after parturition. Each pen allows for 9 m^2^lying space per cow.
Study design	Randomised control trial.
Outcome studied	Behavioural observations began immediately post-parturition, and behavioural data was collected using instantaneous sampling. A 48-hour time budget was constructed for every cow. Postpartum behaviour was analysed every even hour of the first 24 hours, with a sampling interval of every 20 minutes. Behavioural observations were considered in four 12-hour time blocks: 0–12 hours, 12–24 hours, 24–36 hours, and 36–48 hours. ‘Primary’ behaviours (lying behaviours, standing and walking) were recorded during all time points that the cows were visible whereas ‘secondary’ behaviours (social behaviours, grooming behaviours, feeding and drinking directed behaviours) were exhibited concurrently with primary behaviours and were recorded when observed.
Main findings (relevant to PICO question)	Due to technical failure, substantial video footage for some animals was lost, and these animals were not included in behavioural analysis. Thus, 72 cows (rather than 94 cows originally recruited) were included in the final behavioural analysis. It is unclear whether the final sample met the requirements of the power calculation performed. Cows in the NSAID treatment group spent less time in lateral recumbency with their head in a rested position and less time overall in lateral than cows in the placebo group. Additionally, when lying in sternal recumbency, cows in the NSAID group spent less time with the head held in an elevated position and more time with their head in a rested position than cows in the placebo group. The interaction between assistance and treatment status did not significantly affect any behaviours or postures.
Limitations	Blinding to the treatment status was successful but blinding of the observer to assistance status of the subjects was not possible. Cows were recorded as not visible when they were milking, and it is likely that this has caused the proportion of the time budget engaged in standing behaviours to be slightly underestimated. This could have resulted in some behaviours being under-represented. Parturition assistance was provided by one experienced stockperson, and early intervention with parturition assistance was only performed if the calf’s birth was a malpresention. Thus, cows experiencing assisted parturition may not have experienced as much soft tissue trauma and associated pain as cows managed in a less optimal manner. Only parturition events that produced live female Holstein calves were included in this study which may also have minimised the degree of soft tissue trauma experienced by recruited cows. Although instantaneous sampling intervals of 20 min have been shown to represent standing and lying behaviours well, feeding behaviours are better represented by shorter sample intervals. Therefore, it is possible that the proportion of time spent feeding was underestimated.

Table note...

**Mainau et al. (2014):** 

Population	The study took place in Spain between September and December 2008. Cows (from first to sixth parity) from a commercial dairy farm that had 1000 Friesian dairy cows were selected given they had an acceptable body condition score (≥ 2.5 and ≤ 3.5), did not have lameness, and had no other clinical sign of illness. Only calving that required no assistance or assisted calving by means of an easy manual pull applied only by 1 person were studied. Dystocic calvings, stillbirths, cases that required additional anti-inflammatory treatment, and twin calvings were excluded.
Sample Size	60 cows.
Intervention Details	Cows were randomly allocated into 2 groups according to parity and treated with either 0.5 mg/kg meloxicam (Metacam 20 mg/mL solution injection) or 0.5 mg/kg placebo (excipient without pharmacologically active ingredient), injected subcutaneously in the neck. Treatments were administered when cows entered the post-calving pen within a maximum of 6 hours. Twenty-five heifers (12 in the meloxicam group and 13 in the control group) and 35 multiparous cows (17 in the meloxicam group and 18 in the control group) were included. Each cow was studied from 5 days prior to the expected calving date to one month after calving. To ensure blinding, the meloxicam and control groups were labelled A and B, respectively, to ensure blinding of producers and to the treatment and control groups. Approximately five days prior to calving, cows were moved to the pre-calving pen. The pre-calving pen was covered in fresh straw. Once the calf was moved, cows were moved to the post-calving pen. Space allowance per cow was not discussed.
Study design	Blinded randomised control trial.
Outcome studied	During calving, the following events were recorded: calf position, sex of calves and level of calving assistance. Milk production (kg/cow/milking) was recorded for 1 month. Rectal temperature was measured using a single thermometer probe and a consistent penetration depth for all measurements. Rectal temperature was taken when cows were placed in the precalving pen (day -5), twice for 3 days after calving, and every time a blood sample was taken. Just before administration of either treatment or placebo, blood samples were collected from the coccygeal vein of 20 cows (balanced for parity and treatment) to determine serum amyloid A (μg/mL) and haptoglobin (mg/mL) on the day of calving (day 0) and on days +2, +4, and +15 after calving. Cow activity, calculated as the average number of steps per hour over 1 day, was obtained using pedometers from 1 day before to 7 days after calving. Pedometers were placed above the fetlock on the left hind leg of each animal. To monitor cow behaviour throughout the day, 2 video cameras and a digital recorder were installed in each pen. Cow behaviours were observed for 2 days before and after calving. Screening was performed by 2 different observers who were previously trained by individually observing the same 4 cows over 2 days. Every 10 minutes, the cows were classified into the following behaviours: cow posture, location of cows in pen, feeding, tail position, and body behaviours.
Main findings (relevant to PICO question)	Video recording and therefore behaviour data could only be retrieved from 54 cows: 26 from the treatment group (9 heifers and 17 multiparous cows) and 28 from the control group (10 heifers and 18 multiparous cows). Overall activity (average number of steps per hour over a day) was comparable between treatment groups before calving. Heifers from the meloxicam group showed a significantly higher activity during the first 2 days after calving than heifers from the control group. The activity of multiparous cows was comparable in both groups. There was no significant effect of treatment, parity, or calving assistance on postures, changing posture, location in pen, or feeding behaviours.
Limitations	It was unclear whether the sample size was calculated and there was no evidence of 95% confidence intervals to evaluate whether sample size was sufficient. Although treatments were administered within a maximum of 6 hours of entering the post calving pen, administration times would have varied between treatment cows. The scan methodology with a 10-minute interval utilised in this study may not have been sensitive enough to detect changes in eating behaviour.

Table note...

**Swartz et al. (2018):** 

Population	Pregnant dry cows and heifers (Holstein Friesians and Jerseys) that received their respective treatment in the window specified by their treatment group and that did not calve with twins or calve via caesarean. The study was conducted on a Dairy Research Complex in Virginia, USA, and enrollment occurred from August 2016 through August 2017.
Sample Size	194 (87 multiparous Holstein, 48 primiparous Holstein, 41 multiparous Jersey, and 18 primiparous Jersey).
Intervention Details	Holstein and Jersey cows in the study were randomly assigned to one of three treatment groups: Meloxicam administration no more than 48 hours before calving and no less than 6 hours before calving, and a placebo administered within 12 hours postcalving (MEL-PRE, n = 60). Placebo administration no more than 48 hours before calving and no less than 6 hours before calving, and meloxicam administered within 12 hours postcalving (MEL-POST, n = 69). Placebo administration no more than 48 hours before calving and no less than 6 hours before calving, and a placebo administered within 12 hours postcalving (CTL, n = 65). Meloxicam tablets were administered by mouth at 1 mg/kg body weight. Meloxicam tablets were placed into gel capsules and an empty gel capsule was used as a placebo. Farm managers and staff were masked to treatments during calving. Vaginal thermometer was utilised to detect the drop in temperature that occurs 48 hours before calving. Cows and heifers had their temperature taken approximately 14 days before their calving date to aid in detection of the drop in temperature 48 hours prior to calving. When the temperature declined approximately 0.6 °C or more the treatment regimen was initiated. Approximately three weeks before expected calving dates all pregnant dry cows and heifers were moved to group housing in compost-bedded pack barn with access to a close-up dry cow total mixed ration. Following calving, cows were moved into an adjacent compost-bedded pack pen. After the first week of lactation, cows were moved into a deep sand-bedded free stall barn, where they remained for the rest of the trial. A minimum area of 9.3 m^2^ per cow was maintained in both housing areas.
Study design	Blind randomised control trial.
Outcome studied	Cows and heifers were monitored continuously 24 hours/day using 3 video cameras. Dystocia was defined when stage 2 of labour was > 70 minutes and eutocia was defined as < 70 minutes. Animals were fitted with an accelerometer (AfiTag II, AfiMilk Ltd.) that measured activity, lying time, and lying bouts. Data recording began 14 days before the expected calving date and concluded at 7 days in milk (DIM). Rectal temperatures for the first 7 DIM were taken at ~09:00. using a digital rectal thermometer and clinical disease events were diagnosed by two veterinarians.
Main findings (relevant to PICO question)	For the accelerometer behaviour measurements, some cows did not have day -3 data, thus the final sample size for behavioural data included only 165 animals. Lying time: No effect of treatment was identified for lying time. Activity: No treatment effect was found in the eutocic animals. Dystocic MEL-PRE animals were less active than dystocic CTL. Moreover, dystocic MEL-POST animals were also less active than dystocic CTL animals. No difference was noted between the dystocic MEL-PRE and dystocic MEL-POST animals. No differences were found between treatment groups within the Holstein breed. A treatment effect was noted in the Jersey breed whereby Jerseys that where in the MEL-PRE group were significantly less active on d -1, d 1, and d 2 when compared with the Jersey CTL. Additionally, Jersey MEL-PRE were less active than Jersey MEL-POST on d 2. Jersey cattle in the MEL-POST group were less active than Jersey CTL on d 6. Number of lying bouts: Primiparous MEL-PRE animals had more lying bouts on d 0 than primiparous CTL and primiparous MEL-POST. Primiparous CTL had fewer lying bouts on the day of calving than that of all treatment groups of multiparous animals. Similarly, primiparous MEL-POST animals had fewer lying bouts on the day of calving when compared with all treatment groups of multiparous animals. A parity effect was identified on the day before calving, as primiparous MEL-POST animals showed more lying bouts than multiparous MEL-POST. Lying bout duration: No effect of treatment was identified.
Limitations	Sample size calculation was omitted and there was no evidence of 95% confidence intervals to evaluate whether sample size was sufficient. 194 cows were used for analysis after the enrolment of 237 cows due to cows not receiving the precalving treatment within the specified timeframes. The definition of dystocia was defined by calving length, versus intervention status utilised in other studies which may contribute to the significantly different findings between papers.

Table note...

### Appraisal, application and reflection

Five randomised control trials were included in the analysis. Two of the studies did not state whether there was blinding of study personnel (Barragan et al., 2020; Gladden et al., 2021) as the element of observer bias may influence the outcome. Studies by Swartz et al. (2018) and Mainau et al. (2014) omitted calculations to determine the sample size required to deliver sufficient study power and there was no evidence of 95% confidence intervals to evaluate whether their chosen sample size was sufficient. Consequently, some of the non-significant results may be an artefact of low power, limiting the reliability of each study’s conclusions. This may stand for those studies that utilised a small subset of the study animals for the behavioural analysis component, without a sample size calculation to justify the number of individuals within these smaller subsets, the reliability of the results is questionable (Barragan et al., 2020; Gladden et al., 2021). It is important to address that these analyses are of a subjective nature and must be interpreted with caution. Nevertheless, all of the studies had at least one significant finding relating to pain-related behaviour in postpartum cows.

There were no reports of retained placenta or stillbirths associated with NSAID administration in any of these studies. The papers that utilised meloxicam all reported that there was no association between the incidence of adverse outcome post-calving and NSAID administration (Mainau et al., 2014; Swartz et al., 2018). However, the paper that utilised ketoprofen did measure the incidence of these undesirable side-effects, but the outcome was not reported (Gladden et al., 2021).

Studies using flunixin meglumine were excluded from this analysis as cows treated with Flunixin post-calving had an increased risk of retained placentas, still births and metritis (Duffield et al., 2009; Newby et al., 2017). This is due to its propensity to preferentially inhibit cyclooxygenase 1 (COX-1), in contrast to meloxicam which is predominately a cyclooxygenase 2 (COX-2) inhibitor (Trimboli et al., 2020). Thus, it has been recommended that flunixin should not be administered to cattle within 24 hours of parturition (Newby et al., 2017). Ketoprofen’s COX selectivity in cows has not been reported, but it has been reported to lower incidence of retained foetal membranes (Richards et al., 2009). Acetylsalicylic acid has been reported to be a weak inhibitor of both COX isoforms (Barragan et al., 2020). However, the paper that utilised acetylsalicylic acid did not report on the incidence of any of the aforementioned undesirable effects (Barragan et al., 2020).

Lying was reported in all of the papers included in the analysis. When primiparous animals were administered meloxicam prior to natural calving, they displayed significantly more lying bouts on the day of calving when compared to primiparous control animals and primiparous animals that were administered meloxicam postpartum (Swartz et al., 2018). Interestingly, the number of lying bouts in the pre-calving meloxicam group on the day of calving was more similar to the multiparous cows (Swartz et al., 2018). Furthermore, primiparous controls and primiparous animals that received meloxicam postpartum had significantly fewer bouts of lying on the day of calving than all treatment groups of multiparous cows (Swartz et al., 2018). Multiparous animals typically deliver more easily than primiparous animals, but this data suggests that the anti-inflammatory effects of pre-calving meloxicam in primiparous animals allows for an easier calving, or a calving comparable to that of multiparous cows (Swartz et al., 2018). Although these conclusions are not measuring the reduction in a pain-related behaviour and thus are not directly aligned with the research question, the presence of a behaviour associated with the reduction in pain is important to report. The study that administered meloxicam to their treatment animals’ postpartum concluded insignificant lying results (Mainau, 2014). However, significant lying results were also reported in a study by Barrier et al., 2014 which stated that when cows undergoing a caesarean were administered meloxicam prior to the first incision, they spent more time lying and had more bouts of lying within the first 16 hours and 24 hours respectively, when compared to the placebo group (Barrier et al., 2014).

The study by Swartz et al. (2018) relied on the drop in rectal temperature that occurs prior to parturition to aid in calving detection. This method of detection has moderate accuracy (at best) and resulted in 43 cows being removed from the study as they did not receive the pre-calving treatment within the specified timeframe. This demonstrates that the application of administering meloxicam before calving is challenging. Advances in identifying the onset of calving through technology that measures rumination, lying and activity provides promising potential in this space (Borchers et al., 2017; Fadul et al., 2017).

To support the theory that NSAID administration reduces pain postpartum, the Gladden et al. (2021) study analysed specific lying postures found that all cows administered ketoprofen spent less time in lateral recumbency than cows treated with saline, irrespective of whether calving was assisted. Lateral recumbency in adult cattle is considered an abnormal lying posture exhibited when animals are in pain or are unwell (Hudson et al., 2008; Petherick et al., 2014). Therefore, this suggests that pain is experienced after parturition in all cows and ketoprofen administration improves comfort irrespective of parturition experience (Gladden et al., 2021). It is important to recognise that this conclusion is drawn based on the theory that lateral recumbency in cattle is an indicator of pain. Additionally, when treatment cows were in sternal recumbency, they spent more time with their head in a rested position than the placebo group (Gladden et al., 2021). Such posture is usually adopted when in a deep sleep (i.e., rapid eye movement sleep) (Ruckebusch, 1975). It is possible that pain reduced the duration of the placebo group’s sleep, explaining why the placebo group spent less time with their head rested (Gladden et al., 2021; Ruckebusch, 1975). The Gladden et al., 2021 study only included parturition events that produced live dairy female Holstein calves (Gladden et al., 2021). Considering that male dairy calves consistently weigh more than females the selection of only female associated calving events limits the usefulness of this data and its ability to be extrapolated to all calving events (Dhakal et al., 2013; Olson et al., 2009).

Activity measures of the study animals were taken either via the use of accelerometers or pedometers (Barragan et al., 2020; Mainau et al., 2014; Swartz et al., 2018). One study found no significant effects in eutocic animals (Swartz et al., 2018). However, dystocic animals in this study that received meloxicam prior to or after calving were less active than the dystocic control animals postpartum (Swartz et al., 2018). The administration of meloxicam may have reduced activity by alleviating inflammation, allowing cows to rest more easily postpartum (Swartz et al., 2018). Conversely, studies also concluded that cows or heifers treated with NSAID postpartum had increased activity when compared to the control animals and that postpartum administration does lead to a reduction in inflammatory mediators, ameliorating behaviours associated with pain, and allowing these individuals to exhibit their normal behaviours (Barragan et al., 2020; and Mainau et al., 2014). Consequently, activity as a behavioural measure was difficult to analyse due to the conflicting findings presented.

Two of the studies assessed the differences in feeding behaviour postpartum (Gladden et al., 2021; Mainau et al., 2014) . These studies did not find a significant result regarding feeding behaviour. These insignificant results could have been explained by the adoption of scan methodology whereby feeding was measured in 10- or 20-minute intervals. Feeding behaviours are better represented by shorter sample intervals (Gladden et al., 2021; Mainau et al., 2014; Mitlöhner et al., 2001). Thus, it is possible that the proportion of time spent feeding was underestimated as the chosen interval was not sensitive enough to detect a change in feeding behaviour (Gladden et al., 2021; Mainau et al., 2014). Due to the discrepancy in the results reported and the potential that the scan methodology adopted could have had an influence, the need for further exploration into NSAID effect on feeding behaviour postpartum is validated.

Upon critical analysis, NSAID proved to reduce pain-related behaviours or increase resting behaviours in postpartum cows most consistently when they were administered 48 hours prior to but no less than 6 hours before calving, or within three hours of calving (Gladden et al., 2021; Swartz et al., 2018). This conclusion proved to be most consistent in individuals that had experienced uncomplicated calving events, primiparous animals and potentially those that experienced dystocia; however, due to the conflicting analysis of activity between studies, this should be interpreted with caution (Gladden et al., 2021; Swartz et al., 2018). This analysis has revealed that the inflammatory state that has been identified and reported on in postpartum cows which experienced uneventful calving could be dampened down by NSAID administration (Gladden et al., 2021). Additionally, multiparous animals typically deliver calves more easily than primiparous cows, but the literature suggests NSAID administration to primiparous cows in the recommended time frame could allow for a calving experience similar to that of a multiparous cow (Swartz et al., 2018). As a result, if NSAID are introduced into primiparous calving protocols to improve calving ease, not only could the culling rates of animals who experienced calving difficulties be reduced, but animal welfare could be improved (MLA, 2019; Swartz et al., 2018). To explore this further, and due to the low numbers of studies which focuses on NSAID administration to cows that experienced uncomplicated calving events, are primiparous or that experienced dystocia more research is warranted.

Interestingly, two included studies measured milk yield postpartum and found that milk-yield was greater in the cows administered NSAID when compared to those that received a placebo (Barragan et al., 2020; Swartz et al., 2018). It cannot be denied that administration of NSAID to cows is laborious for producers but the increase in milk-yield may provide them with some incentive to consider administering NSAID to cows approaching parturition or promptly post-parturition.

### Methodology

**Search strategy:** 

Databases searched and dates covered	CAB Abstracts Web of Science Core Collection from 1910 to 31 January 2024 Medline via OVID SP from 1946 to 31 January 2024
Search terms	CAB Abstracts: TS=(Cows OR Cattle NOT calves) AND TS=(behaviour OR behavior) AND TS=(Parturition) AND TS=(Flunixin OR Ketoprofen OR meloxicam OR NSAID OR Tolfenamic acid OR acetylsalicylic acid OR aspirin) Medline: (cows.mp. or cattle/) and (behaviour.mp. or behavior/) and (parturition/) and (flunixin.mp. or ketoprofen/ or meloxicam/ or anti-inflammatory agents, non-steroidal/ or tolfenamic acid.mp. or acetylsalicylic acid.mp. or aspirin/)
Dates searches performed:	31 Jan 2024

Table note placeholder

**Exclusion / inclusion criteria:** 

Exclusion	Systematic reviews or meta-analyses of randomised control studies, review articles, conference proceedings, case reports/studies that are irrelevant to the PICO.
Inclusion	Studies must contain information that is relevant to the PICO question, and they must be randomised control trials.

Table note placeholder

**Search outcome:** 

Database	Number of results	Excluded – Systematic reviews and meta-analysis of RCTs	Excluded – Review Articles	Excluded – Conference proceedings	Excluded – Case report/ study	Excluded – irrelevant to PICO	Total relevant papers
CABAbstracts	18	1	0	0	0	13	4
Medline	9	0	0	0	0	6	3
Total relevant papers when duplicates removed							4

Table note placeholder

### ORCiD

Amelia Cannadine: https://orcid.org/0000-0003-0147-6601

### Conflict of interest

The author declares no conflicts of interest.

## Disclaimer

Knowledge Summaries are a peer-reviewed article type which aims to answer a clinical question based on the best available current evidence. It does not override the responsibility of the practitioner. Informed decisions should be made by considering such factors as individual clinical expertise and judgement along with patient’s circumstances and owners’ values. Knowledge Summaries are a resource to help inform and any opinions expressed within the Knowledge Summaries are the author’s own and do not necessarily reflect the view of the RCVS Knowledge. Authors are responsible for the accuracy of the content. While the Editor and Publisher believe that all content herein are in accord with current recommendations and practice at the time of publication, they accept no legal responsibility for any errors or omissions, and make no warranty, express or implied, with respect to material contained within. For further information please refer to our Terms of Use.
